# First Epigravettian Ceramic Figurines from Europe (Vela Spila, Croatia)

**DOI:** 10.1371/journal.pone.0041437

**Published:** 2012-07-24

**Authors:** Rebecca Farbstein, Dinko Radić, Dejana Brajković, Preston T. Miracle

**Affiliations:** 1 McDonald Institute for Archaeological Research, University of Cambridge, Cambridge, United Kingdom; 2 Centre of Culture, Archaeological Collection, Vela Luka, Croatia; 3 Institute for Quaternary Paleontology and Geology, Croatian Academy of Sciences and Arts, Zagreb, Croatia; 4 Department of Archaeology, University of Cambridge, Cambridge, United Kingdom; University of Oxford, United Kingdom

## Abstract

Recent finds of 36 ceramic artifacts from the archaeological site of Vela Spila, Croatia, offer the first evidence of ceramic figurative art in late Upper Palaeolithic Europe, *c.* 17,500–15,000 years before present (BP). The size and diversity of this artistic ceramic assemblage indicate the emergence of a social tradition, rather than more ephemeral experimentation with a new material. Vela Spila ceramics offer compelling technological and stylistic comparisons with the only other evidence of a developed Palaeolithic ceramic tradition found at the sites of Pavlov I and Dolní Věstonice I, in the Czech Republic, *c.* 31,000–27,000 cal BP. Because of the 10,000-year gap between the two assemblages, the Vela Spila ceramics are interpreted as evidence of an independent invention of this technology. Consequently, these artifacts provide evidence of a new social context in which ceramics developed and were used to make art in the Upper Palaeolithic.

## Introduction

The Upper Palaeolithic preserves a rich and diverse record of early technological innovations, including textiles and weaving [1], complex organic (bone, ivory and antler) tools, and the earliest undisputed figurative art [2]. One of the most famous Palaeolithic innovations is the ceramic technology that was used to make figurative art at Gravettian (Pavlovian) sites in Moravia, Czech Republic, *c.* 31,000–27,000 cal BP [3]. Until recently, these figurines were among the rare examples of ceramic technology pre-dating the earliest pottery, which has been found at Jomon sites in Japan, *c.* 12,000 cal BP and in late Palaeolithic-aged contexts in China [4]. Thirty-six ceramic figurines and fragments were recently excavated from the archaeological site of Vela Spila, Croatia. These new discoveries, which date to *c.* 17,500–15,000 cal BP, offer the first and only evidence of ceramic figurative art in southeastern Europe during the Upper Palaeolithic. The size, diversity, and complexity of this ceramic art assemblage indicate the emergence of a social tradition rather than more ephemeral experimentation with a new material. On morphological, technological and stylistic grounds, Vela Spila ceramics offer compelling comparisons with the only other evidence of a developed Palaeolithic ceramic tradition, found at Pavlovian sites, such as Pavlov I and Dolní Věstonice I, in the Czech Republic. The new finds from Vela Spila, like the Pavlovian ceramics, provide insight into how socio-technical innovations developed, were adopted, and were sometimes rejected from Upper Palaeolithic socio-technical and artistic repertoires. On current evidence, ceramic technologies seem to have been independently invented *c.* 17,500 years ago, and were subsequently lost from the socio-technical tradition at this site between about 2,000 and 3,000 years later. Consequently, these artifacts provide evidence of a new location and context in which ceramics developed and were used to make art in the Upper Palaeolithic. They encourage consideration of broader archaeological concerns such as the social role of experimentation and innovation and the impact of technological innovations on artistic expression.

## Materials and Methods

**Figure 1:**
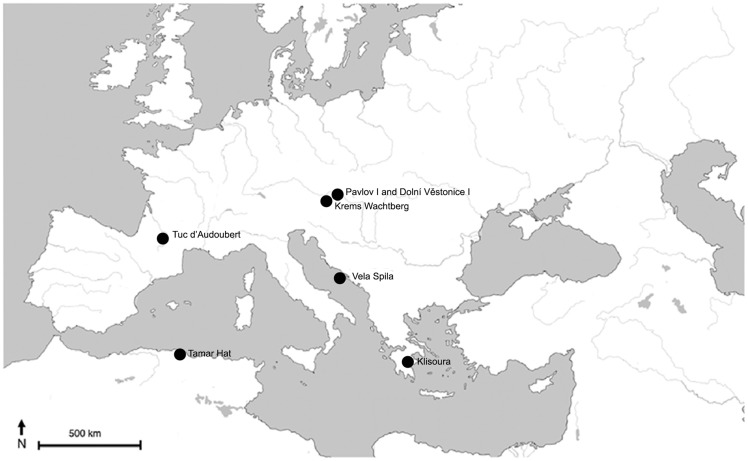
The location of Vela Spila, Croatia and other European sites with Palaeolithic ceramic technologies.

**Figure 2:**
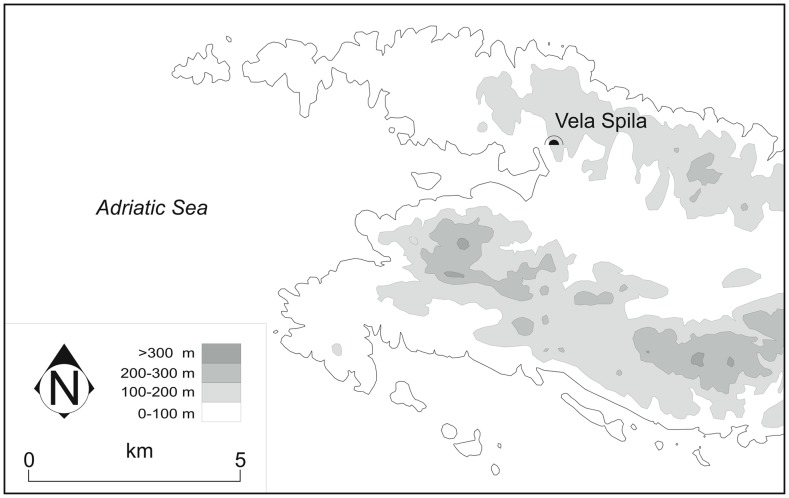
Detailed map of the western end of Korčula island, with the location of Vela Spila marked.

Vela Spila is a cave on the western end of Korčula island, in the central Dalmatian archipelago, Croatia (Figures 1 & 2). The first archaeological excavations were conducted in 1951. Fieldwork continued under the supervision of Božidar Čečuk (1974–1995), Dinko Radić (1996–2006), and Dinko Radić and Preston Miracle (2007– present). Vela Spila preserves evidence of occupation from the Late Upper Palaeolithic (Epigravettian) through the Bronze Age. This paper focuses on ceramic artifacts excavated from Epigravettian contexts in 2001 and 2006. All necessary permits were obtained for the described field studies. Permits were obtained for the excavations from the Ministry of Culture, Republic of Croatia, and no permits were required for the post-excavation analyses of the materials.

**Figure 3:**
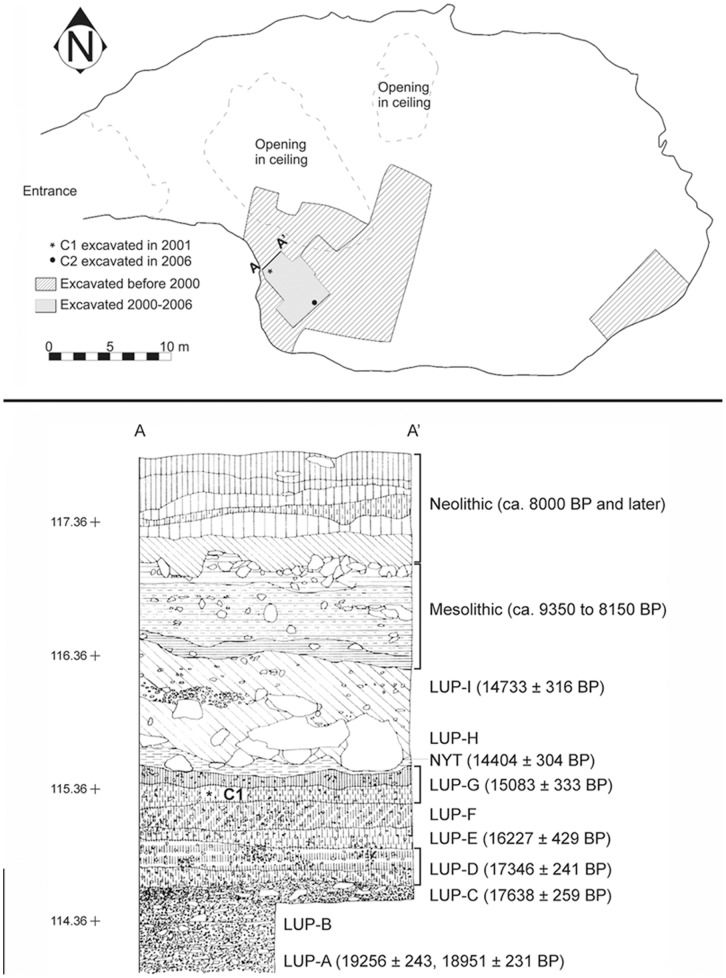
Plan map and stratigraphic profile of Vela Spila. At top, the plan drawing of Vela Spila, with excavated areas highlighted and the approximate find location of C1 and C2 marked. Below, the stratigraphic profile corresponding with the profile marked in the plan drawing. Archaeological horizons, calibrated radiocarbon dates, and the stratigraphic position of C1 are noted.

Vela Spila consists of a single, large chamber approximately 50 m long, 30 wide, and 17 m high (Figure 3). The cave formed in Cretaceous (Cenomanian) limestones that are strongly cracked, weathered, recrystallized, and show different degrees of dolomitization. The cave’s entrance (4 m wide×10 m high at 121 m asl) faces toward the southwest and overlooks Vela Luka Bay. At the time of the Upper Palaeolithic use of the cave, the bay would have been exposed land due to the lowering of sea levels during the Pleistocene (up to −120 m) and the coastline would have been about 10 km away. Today, the cave interior is relatively well lit as it receives natural light through two holes in the cave ceiling (11×9 m and 5×4 m) as well as through the entrance. The age of the opening of these holes in the ceiling is currently unknown.

Pleistocene sediments have been systematically excavated in an area of about 20 square meters (Figure 3). Sediment excavated in 2001 and 2004 (10 square meters) was dry sieved using a 5 mm mesh, while all sediment excavated in 2006 (10 square meters) was wet sieved using a 3 mm mesh. A four meter sequence of Pleistocene sediments has been exposed (Figure 3); bedrock has not been reached. The lowest unit (1 meter thick) does not contain any remains and was deposited before *c.* 20,000 cal BP, probably at the time of the Last Glacial Maximum. Overlying it is a series of layers with abundant archaeological remains; radiocarbon assays on charcoal and bone date these deposits from *c.* 19,500–14,500 cal BP. These contain abundant lithic and organic Upper Palaeolithic remains that are typologically similar to those from well-known Epigravettian sites in the wider region (e.g. Crvena Stijena, Badanj, Kopačina, Šandalja, Grotta Paglicci, etc.). Large vertebrate faunal remains are extremely frequent and are dominated by red deer (*Cervus elaphus*), followed by the extinct half-ass (*Equus* cf. *hydruntinus*); other taxa either are relatively rare (i.e. roe deer [*Capreolus capreolus*], aurochs/bison [*Bos/Bison*], wild boar [*Sus scrofa*], hare [*Lepus* sp.]) or are represented by only a few specimens (i.e. wolf [*Canis lupus*], lynx [*Felis lynx*], wild cat [*Felix silvestris*], fox [*Vulpes vulpes*], hedgehog [*Erinaceus* sp.]) [5]. Smaller vertebrate remains (e.g. rodents, birds, bats, reptiles, fish) are not abundant in the Pleistocene layers.

The ceramics were found during wet-sieving of finds post-excavation in 2001 and 2006. All ceramics are attributable to one of the Late Upper Palaeolithic (LUP) horizons: LUP-D, E, F, or G. As noted above, artifacts and faunal remains found in LUP-D, E, F and G are broadly consistent with nearby regional Epigravettian sites [6]. A series of standard and AMS radiocarbon dates have been processed from LUP-D, E, F and G horizons at Vela Spila (Figure 3 and Table 1), yielding calibrated dates between *c.* 17,500 and *c.* 15,000 years ago. These dates confirm the Epigravettian attribution suggested by material culture in these horizons.

**Table 1:** Radiocarbon dates for the Late Upper Palaeolithic archaeological horizons at Vela Spila, with symbolic material culture from horizons with ceramics noted.

Archaeological Horizon	14 C Sample	Material	Method	14 C bp	Calibrated Date (CalPal 8/2009)	# ofCeramics	Wt. of ceramics(g)	Ave. Length of ceramics (mm)	Symbolic Material Culture
Tephra (Neapolitan Yellow Tuff)	VERA 2345	Bone	AMS	12290±40	14404±304	0	0	0	
Late Upper Palaeolithic-G	z-3989	Charcoal	Standard	12700±100	15083±333	6	11.5	19.3	C1; perforated *Luria* sp. *s*hell
Late Upper Palaeolithic-F	None	None	None	None	None	11	20.1	18.3	Ceramics, perforated red deer canines
Late Upper Palaeolithic-E	z-3991	Charcoal	Standard	13300±100	16227±429	5	6.7	17.8	Ceramics, perforated red deer canines
Late Upper Palaeolithic-D	z-3992	Charcoal	Standard	14100±100	17346±241	13	35.2	20.2	C2; perforated shells, perforated red deer canine
Late Upper Palaeolithic-C	z-3993	Charcoal	Standard	14500±100	17638±231	0	0	0	
Late Upper Palaeolithic-A	VERA-2339	Bone	AMS	15690±70	18951±231	0	0	0	
	VERA-2338	Charcoal	AMS	16140±60	19256±243				

These Late Upper Palaeolithic horizons did not contain any clearly intrusive material from later deposits (e.g. Neolithic ceramics or bones of domestic animals) which were also excavated in this area of the cave. The Epigravettian horizons are more than 1.5 m beneath Neolithic contexts with ceramics, and this part of the cave contains no evidence of later prehistoric pits or postholes dug into underlying sediments. Therefore, we can be confident that the Epigravettian ceramics come from secure contexts and are not intrusive from younger deposits.

One ceramic zoomorphic figurine was identified in 2004 during post-excavation analysis of material recovered during the 2001 excavations. The figurine may have been overlooked during the initial sorting of materials in 2001 due to the incongruity of finding a ceramic artifact in Upper Palaeolithic horizons. Owing to this discovery, assemblages excavated in 2006 were carefully examined with the intent of recovering more ceramic fragments; that year, 45 additional fragments were excavated from Epigravettian horizons and identified as potential ceramic figurines and portions of figurines. These artifacts were arbitrarily numbered C1–C46. Beginning in 2010, these objects were analyzed macroscopically, using a hand lens with 10× magnification, looking for evidence of human modification, such as finger pinches, surface engravings, or marks of smoothing, to determine whether fragments are ceramics demonstrating evidence of human modification. All artifacts were measured in three dimensions using digital calibers, and their dimensions were recorded to the nearest 0.5 mm. They were subsequently weighed using jewelers scales, with weight recorded to the nearest 0.1 g. The general shape (cylinder, dome, round, conical, tabular, irregular, or figurative) and color (tan, tan-brown, orange-brown, brown, or white-grey) were recorded for each object. The presence of surface incisions, puncture marks, irregularities of the surface, impressions, and breaks was noted. Evidence of modeling, for instance joins, pinch marks, or rolling, was recorded. An overall, qualitative description was also recorded. Finally, each artifact was photographed from all angles, using a tripod-mounted digital SLR camera.

**Figure 4:**
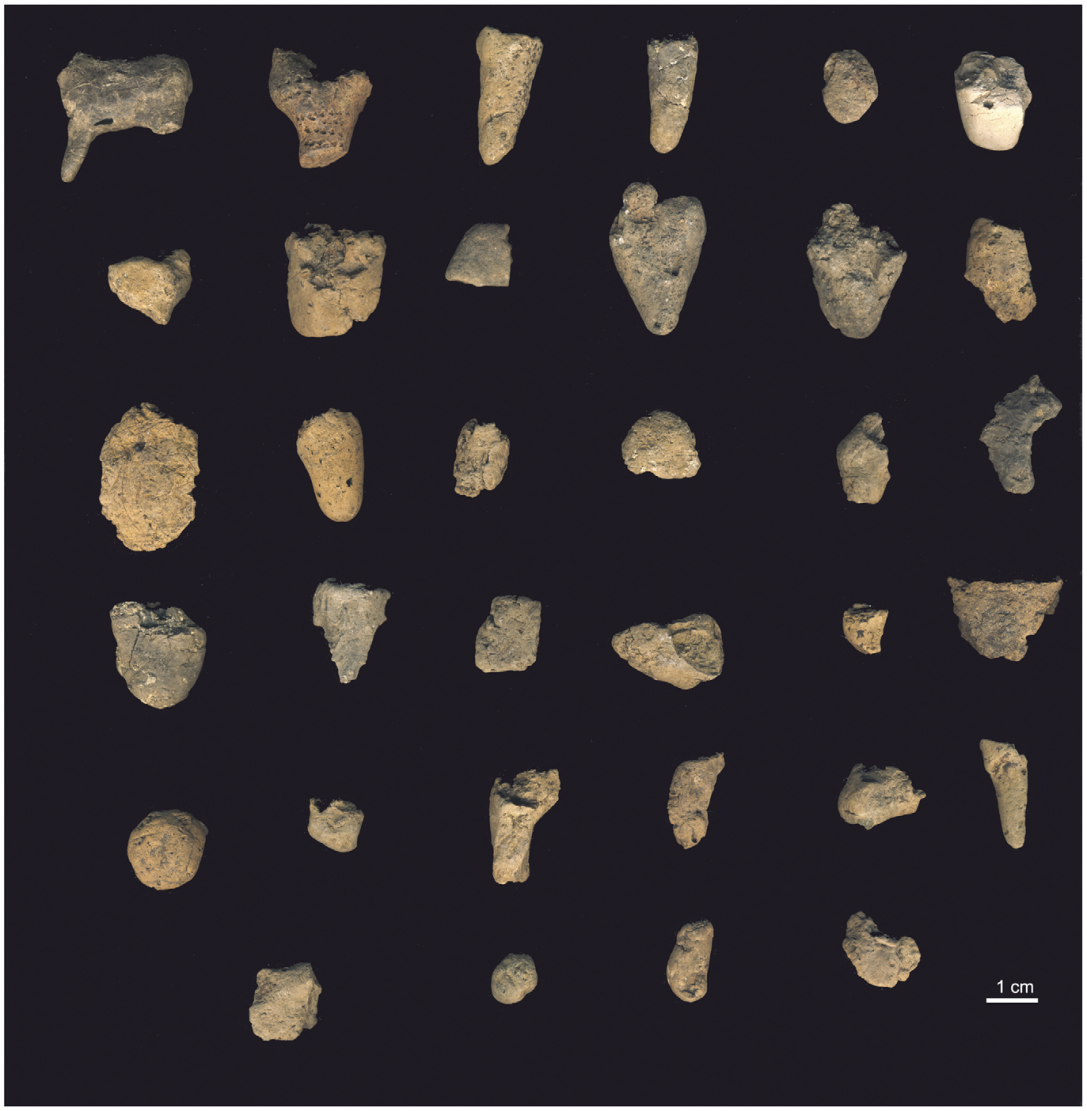
The assemblage of 36 ceramics artifacts from Vela Spila. From top (L to R): C1, C2, C3, C4, C5, C6; C7, C8, C9, C10, C12, C13; C15, C17, C18, C19, C22, C23; C25, C26, C27, C28, C29, C30; C32, C33, C34, C35, C36, C38; C40, C41, C42, C44.

From this assemblage of potential ceramic artifacts, ten fragments were removed from the assemblage and reclassified as: ochre or other pigmented mineral (n = 2), calcite accumulation (n = 3), and unintentionally burned or hardened earth that lacks evidence of intentional human modification (n = 5). Thus, 36 objects from the site are accepted as human-made ceramic artifacts (Figure 4). Thirteen of the ceramics are associated with archaeological horizon LUP-D, five with LUP-E, twelve with LUP-F, and six with LUP-G. These objects have a maximum dimension of between 9.0–30.0 mm, and weigh between 0.5–7.6 g.

## Results

The objects were distinguished based on overall color. This is similar to classifications made by Soffer and Vandiver [7], [8] in their analyses of Pavlovian ceramics. Following observations made in the analysis of later prehistoric pottery [9], the color of ceramics indicates numerous factors, including the chemical composition of clay, the atmosphere in which it was fired, and iron and organic materials contents. Variable or mottled colors may indicate a firing atmosphere that is not consistent or uniform. Vela Spila ceramics are orange-tan (n = 10), tan-brown (n = 10), orange-brown (n = 8), brown (n = 7), and white-grey (n = 1).

Tests of durability similar to those described by Soffer and Vandiver [7], [8], [10], in which they submerged samples in boiling and standing water for various lengths of time, were not conducted because they risked damaging or destroying the limited assemblage of artifacts. However, Soffer and Vandiver noted that the darker-colored and orange samples from Pavlov I were more durable and thus represent the upper limit of firing temperature. We hypothesize, by extension, that the orange-brown and dark brown artifacts from Vela Spila (n = 25) correspond to higher firing temperatures, which Soffer and Vandiver have estimated to be 600–800°C for the Pavlovian ceramics. Lighter orange-tan and white-grey objects (n = 11) may not have been fired, or may have been fired at lower temperatures. Alternatively, these differences in color may reflect variability in material, with the lighter and white artifacts having a lower iron content. Sampling the ceramic artifacts to confirm their clay and mineral content is forthcoming.

**Figure 5:**
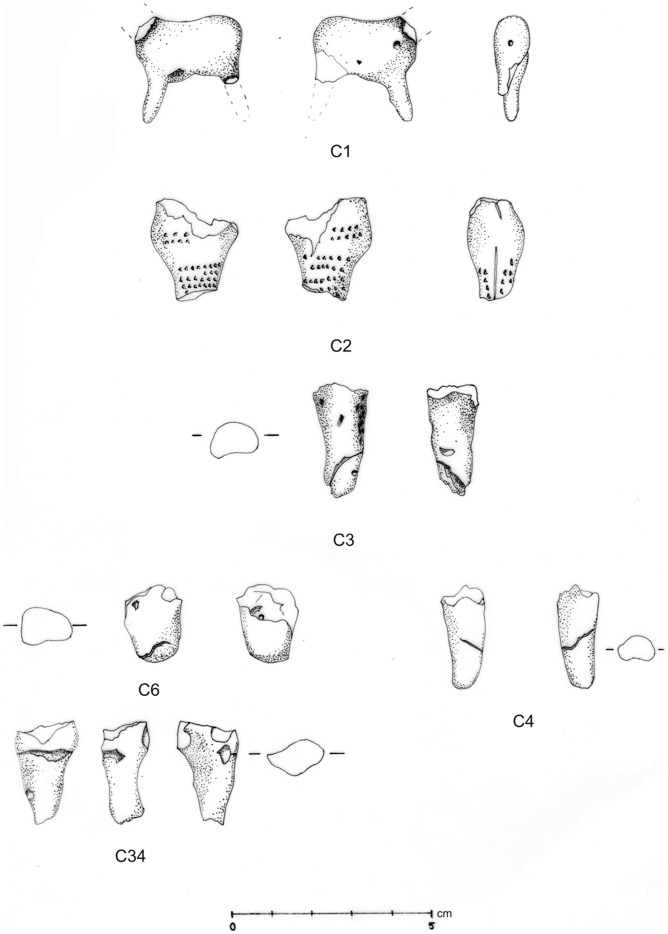
Line drawing of C1, C2, and purported limb fragments from Vela Spila.

**Figure 6:**
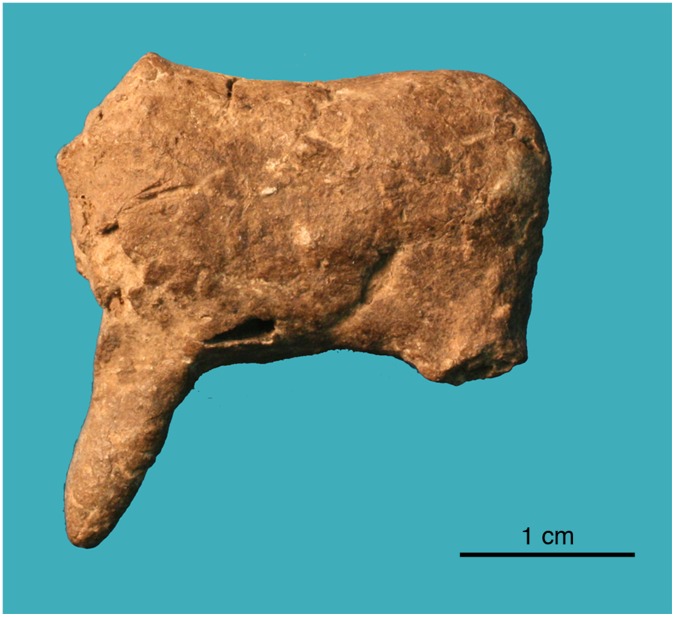
Photograph of C1.

**Figure 7:**
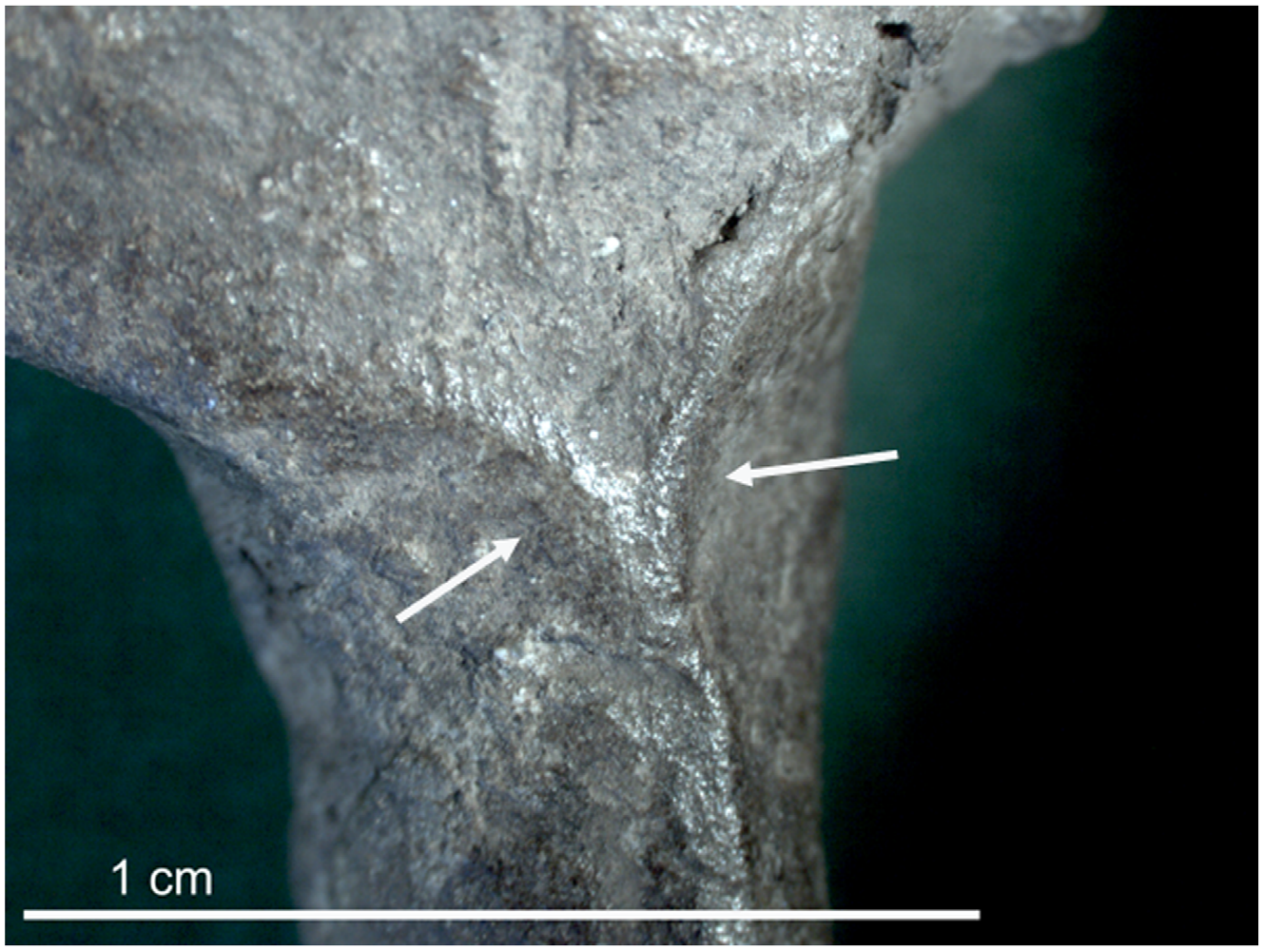
Microscopic photograph of the limb of C1, with pinch marks visible between the arrows.

The most complete artifact, designated C1, was identified in 2004 during analysis of artifacts that were excavated during the 2001 season. C1 preserves the torso and foreleg of an animal, perhaps a horse or deer (Figures 5 and 6). The artifact weighs 4.0 grams, and measures 26.0×27.0×9.0 mm. Its excavation context is an excavation layer labeled “12 B (8/4),” which corresponds with Late Upper Palaeolithic Horizon G (LUP-G) (see Figure 3). Five additional ceramics were subsequently found in horizon LUP-G in 2006. C1 is dark brown and has a smooth texture, suggesting it was fired at a relatively high temperature. The head and hindleg have broken off. Pinch marks are visible under the microscope (Figure 7), suggesting individual body parts were molded separately before being joined together.

**Figure 8:**
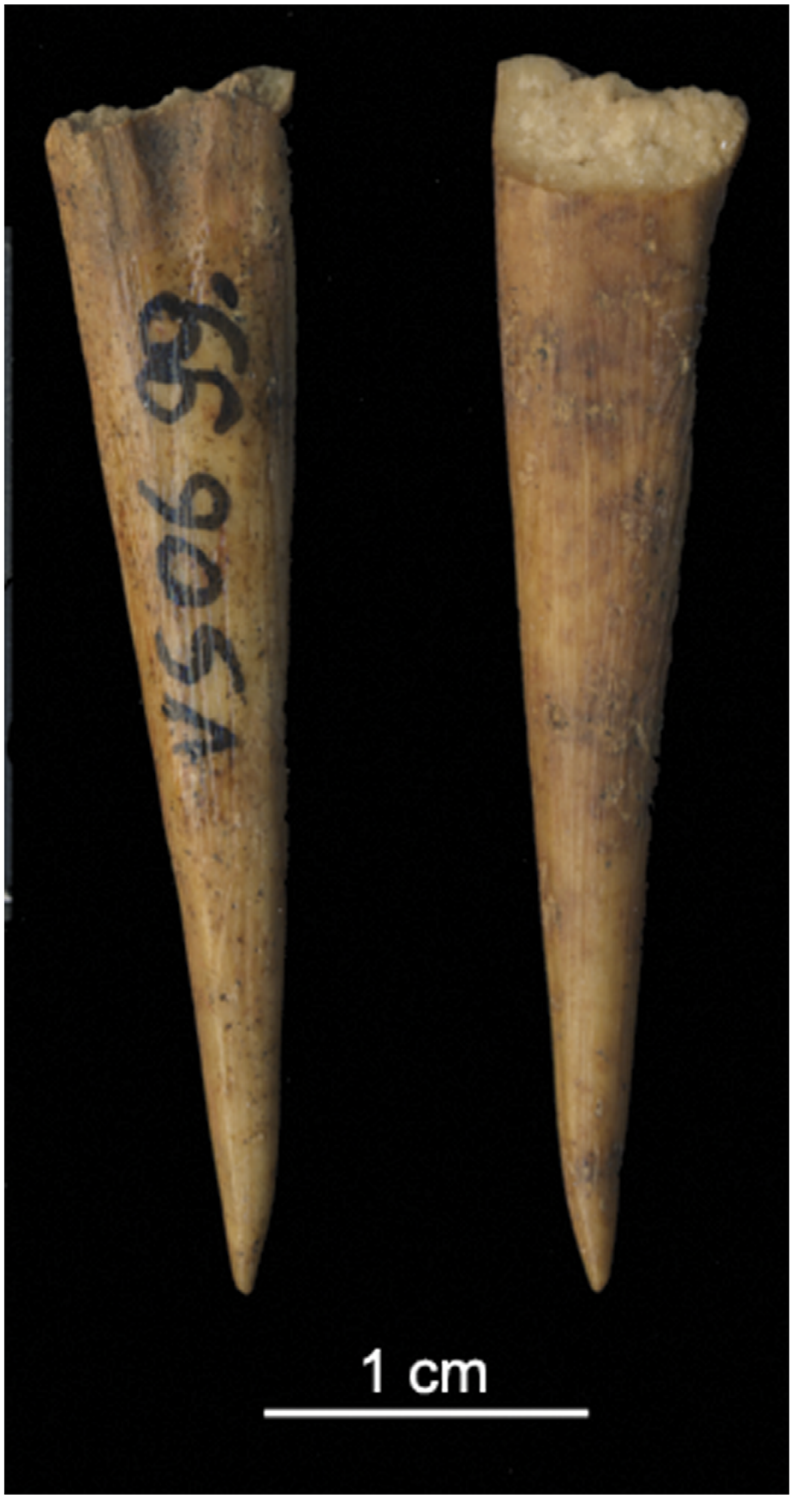
Bone point from LUP-G horizon at Vela Spila (VS 06.99). A bone point similar to this may have been used to create the hole in the C1 figurine.

The figurine preserves no engravings such as facial features, fur or hair, or another decorative marks or patterns. The few faint marks on the torso were likely made unintentionally with a fingernail while the object was being modeled. A hole perforates the animal’s rear, in the anatomical position of the anus. The smooth-edged and uniform hole, measuring 1.67×1.78 mm, was probably made with a tool such as a small bone point. One bone point fragment, VS 06.99, which was excavated in 2006 from Square 12, Layer 20 (within the LUP-G horizon), measures 34.24 mm long and its rounded point tapers to less than 1.1 mm across (Figure 8). This tool fits into the hole in C1. Although we do not purport that VS 06.99 was the point used to make the hole in C1, it demonstrates the presence of bone points in the Late Upper Palaeolithic assemblage at Vela Spila that are an appropriate size to make such a hole. Several other similarly sized bone points were found in horizon LUP-D. It is not possible to discern how deeply the hole on C1 extends into the body, but a second hole on the side of the animal’s torso may indicate where the tool punctured through the torso. A similar hole was noted on the rounded edge of a cone-shaped, tan-brown colored fragment, C10, which was found in the LUP-D horizon.

The surviving foreleg on C1 represents both forelimbs as a single appendage. The artist modeled the two forelegs (as well as the hindlegs, as indicated by the single break) as a single limb. This convention allows the artist to make fewer potentially fragile limbs, thus minimizing the number of joins and imparting more structural strength to the figurine with resulting limbs that are thicker near the join with the torso.

**Figure 9:**
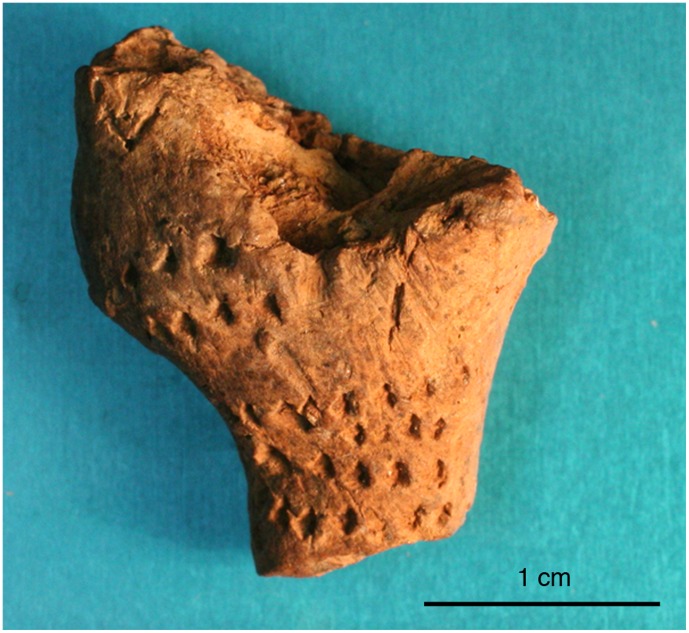
Photograph of C2.

A second figurative fragment, C2 (Figures 5 and 9), was excavated from a layer within horizon LUP-D, radiocarbon dated to *c.* 17,300 cal BP. The piece is fragmented and broken at two extremities, making it difficult to discern the original shape. The fragment is relatively large in comparison to the rest of the assemblage, measuring 25.0×21.0×8.0 mm and weighing 5.0 grams. The dark brown-orange color and smooth texture of this piece are consistent with firing at a reasonably high temperature.

This piece is heavily engraved with bands of incisions which resemble punctures and short hatches. Microscopically, these incisions are visibly V-shaped, suggesting they were made with a burin or the corner of a lithic blade or flake fragment. There is also one shallow engraving or scraping which is internally striated (visible under 10× magnification) extending down one side of the fragment (see the third view of C2 in Figure 5). This mark differs from the incisions that cover the rest of the object as it is shallow, internally striated, and not v-shaped, which suggests it may have been made by a different gesture or tool. Similar striated marks were noted on two other pieces: C12, a conical-shaped, brown fragment with several incisions that are internally striated under 10× magnification, and C15, an orange-tan, tabular or slab-shaped fragment that also preserves several of these striated incisions.

C2 might represent an animal’s hindquarters. The tapered end suggests part of a leg, while the larger portion would be the rear of the animal. If the convention of consolidating limbs was adopted on this piece, as on C1, the preserved fragment would represent both hindlegs. The shallow engraving visually differentiates two legs without physically forming two separate limbs. If C2 is the fragmented hindquarters of an originally complete zoomorphic figurine, the size, shape and proportions of the C2 fragment imply that the complete figurine comprised in part of the C2 fragment would have been larger than C1.

Six cylindrical or conically shaped artifacts (C3, C4, C6, C17, C34, C38) (Figures 4 and 5) suggest limbs similar to the one preserved on C1. All pieces are broken at one extremity, while the other extremity is unbroken and smoothed to a rounded tip. Artifact C38 (Figure 4) resembles the preserved limb on C1, although it is longer and wider than the leg on C1 and does not refit with C1. The other five possible leg fragments are wider and more robust, and several do not taper towards the unbroken tip. No fragment depicts a foot, nor does the preserved foreleg of C1.

**Figure 10:**
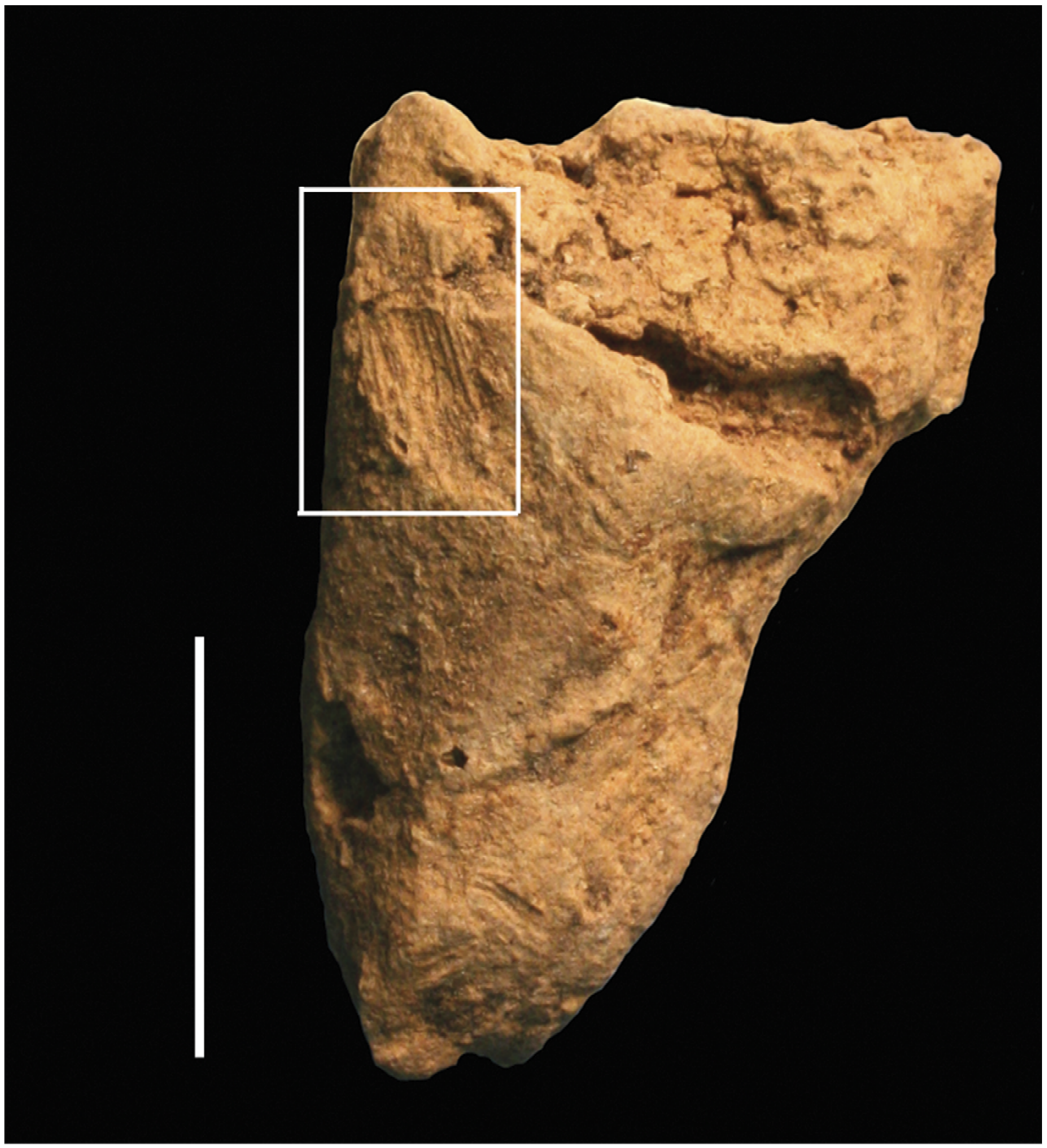
Photograph of C34, with the finger impressions highlighted in the white box.

Two fragments offer primary evidence of human manipulation of ceramics at Vela Spila (C34 and C18). Small impressions or striations are visible with a hand lens on the smoothed surfaces of both objects [Figure 10]. The size and pattern of the striations resemble finger impressions similar to those previously identified on other Palaeolithic ceramics [11], [12]. Wet ceramic pastes formed by modelling with hands would have inevitably collected finger impressions during production, so it is not surprising that several objects in the Vela Spila assemblage preserve such marks.

Other ceramic fragments excavated from Epigravettian horizons at Vela Spila include: C5, an orange, flattened, oval-shaped ceramic; C7, a spherical, orange-brown artifact with one pinched point, with a maximum dimension of 15.0 mm; C8, a tabular, orange-brown artifact with a maximum dimension of 23.0 mm; C9, an orange-brown, dome-shaped artifact with a maximum dimension of 21.0 mm, comprised of two fragments that refit together; C13, a cylindrical, tan-brown artifact with a worked, rounded extremity and a maximum dimension of 21.0 mm; C19, a rounded tabular, orange-brown artifact that appears to have been pinched into shape; C22, an irregularly shaped, tan-brown ceramic with a maximum dimension of 19.0 mm; C23, a brown, cylindrical artifact shaped by rolling and pinching; C25, a cylindrical, dark-brown artifact with one broken extremity and evidence of smoothing across its surfaces; C26, a flattened, triangular shaped fragment with several striations on one surface; C27, a small, tan, tabular shaped artifact, with a maximum dimension of 15.0 mm; C28, a conical, orange-tan artifact with a maximum dimension of 21.0 mm; C29, an orange-tan, cylindrical artifact with one extremity that has been pinched into shape; C30, an orange-brown, irregularly-shaped artifact with a maximum dimension of 23.0 mm; C32, a dome-shaped, orange-tan artifact; C33, a round, tan-brown artifact with round and smoothed surfaces and one broken edge; C35, a small, orange-tan, cylindrical artifact without any breaks at its extremities; C36, a small, conical, brown fragment with a break at one end and a group of incisions near the tapered point; C40, a tabular, tan-brown artifact with a maximum dimension of 15.0 mm; C41, a regular, smoothed, spherical, tan-brown artifact; C42, an orange-brown, cylindrical artifact, seemingly shaped by rolling, featuring one incised mark on its surface; and C44, a flattened, semi-circular shaped artifact with smoothed surfaces (Figure 4).

The ceramic artifacts are important components of the classifiable Epigravettian “art” from Vela Spila. To date, 29 other symbolic or ornamental artifacts have also been found. Perforated shells of *Cyclope* sp., *Luria* sp., and *Lucinidae* sp. were excavated from LUP horizons D-G. Eleven perforated red deer canines, one engraved with six linear incisions, were also excavated from Epigravettian layers. One perforated bone fragment, possibly an ornament or pendant, and two decorated bone tools were also found [6] (Table 1).

## Discussion

The most immediate points of comparison to the Vela Spila ceramics are Pavlovian ceramic assemblages from Moravia, Czech Republic, particularly Dolní Věstonice I and Pavlov I, dated to *c.* 31,000–27,000 cal BP [13] (Figure 1 and Table 2). Pavlovian ceramics were made from loess paste that was fired in hearths at temperatures between 300–700°C [14]. Although these assemblages are separated by more than 10,000 years, there are several important similarities.

**Table 2:** Comparisons between Pavlovian and Vela Spila ceramics.

	Vela Spila	Pavlovian
Zoomorphic depictions	+	+
Anthropomorphic depictions		+
Engraved surfaces	+	+
Broken leg fragments	+	+
“Compressed” legs on animal figurines	+	+
Range of firing temperatures	+	+
Additive technology	+	+
Association with hearths		+
Association with other figurative art		+
Association with ornaments and decorative art	+	+
Legs depicted without feet	+	+
Legs depicted with schematic feet		+

The Vela Spila ceramics, like many Pavlovian ceramics [3], [7], [8], [10], [14], were made by additive processes. Techno-stylistically, C1 resembles several Pavlovian figurines with limbs consolidated or compressed to form a single appendage. C2 recalls Pavlovian ceramics, including the famous Venus of Dolní Věstonice, with an incised line along a single compressed limb, suggesting the presence of two limbs. If C2 is a fragment of a zoomorphic figurine’s hindquarters, it echoes this tradition. The incisions that cover C2 are reminiscent of impressions or punctures found on several ceramics from Pavlov I, including the torso of one female figurine and one of the so-called ceramic “biconical heads.” The many fragments from Vela Spila resemble the hundreds of fragmented limbs found at Dolní Věstonice I and Pavlov I. The finger impressions on ceramics from Vela Spila are also consistent with the Pavlovian record, as several Pavlovian ceramics, including the Venus of Dolní Věstonice, preserve finger impressions [11], [12].

However, the Vela Spila assemblage is distinguishable from the Pavlovian assemblage in several ways. Many ceramic leg fragments from Pavlov I depict a schematic foot [8], [10]. No comparable depiction is apparent at Vela Spila, suggesting a different stylistic convention for depicting zoomorphic legs. Statistical confirmation of this difference between Vela Spila and Pavlov I would require a larger sample size. Additionally, Vela Spila ceramics are the only example of figurative art at the site. In contrast, both figurative and non-figurative art were made in a variety of materials at Pavlovian sites [15], [16].

Vela Spila ceramics do not cluster near settlement features, hearths or concentrations of burned material. Combustion features, including hearths, were excavated in Horizon LUP-H, but this horizon did not contain ceramics. Combustion features were not identified in any of the horizons with ceramics, although all of these horizons yielded both wood charcoal and burned faunal remains (13.9–31.1% of fragments). Dispersed ash was found in some layers where ceramics were also found, but this ash was not associated with rubified sediment which would suggest the presence of *in situ* hearths. Furthermore, because all of the ceramics were recovered in wet-sieved residues rather than in the trench, it is impossible to associate them with any features that might have been present. In contast, Pavlovian ceramics are strongly spatially associated with hearths and purported “kilns” [3], [7], [8], [10], [14]. Some scholars propose that the ceramics had very little “life history” after production [17], which may have included their intentional explosion during firing. The apparently wider distribution of ceramics at Vela Spila suggests that this hypothesized ritualistic destruction of ceramics and strong spatial association with hearths may not have existed at Vela Spila.

Ceramic artifacts from Upper Palaeolithic levels are rare but not unknown beyond the Moravian Gravettian record. A small number of Gravettian ceramics were discovered at Krems-Wachtberg in Austria [18]. Krems-Wachtberg is widely accepted as culturally related to Pavlovian sites in Moravia, and a radiocarbon date of 27,400±300****BP [18] (32,437-31,157 cal BP, calibrated using OxCal v4.1.7) confirms the contemporaneity of these sites. Thus, these finds are likely related to the Pavlovian ceramic socio-technical tradition. A ceramic anthropomorphic figurine was found at Maina in southern Siberia, associated with radiocarbon dates between 16,540±170 BP and 16,176±180 BP [19] (20,221-18,863 cal BP, OxCal v4.1.7). The geographic distance separating Maina and Vela Spila, as well as the stylistic difference between the flattened, silhouetted figurine from Maina and the more rounded objects from Vela Spila, suggest these traditions probably developed independently. Furthermore, based on currently available published materials, the Maina figurine does not seem to have been found alongside a larger assemblage of ceramic fragments, suggesting a different, perhaps more limited, experimentation with ceramic at this site. A similarly isolated Pleistocene ceramic, purported to be a fragment of an animal horn, was found at Tamar Hat, Algeria. The fragment is associated with Iberomaurusian horizons radiocarbon dated to 20,600±500 and 19,800±500 BP [20] (26,007−22,441 cal BP, OxCal v4.1.7). Like the Maina figurine, the fragment from Tamar Hat does not seem to have been found alongside a developed ceramic technological tradition. The few ceramic figurines found in Magdalenian contexts in France [21] might also be best considered isolated experiments. The large, unmoveable and unfired clay statues discovered in Tuc d’Audoubert and Montespan caves (France), which date to the late Magdalenian [21], seem to represent an entirely different tradition and context of production and display.

In other Palaeolithic contexts, ceramic technologies distinguishable from an art tradition have been uncovered. Ceramic hearths found in late Aurignacian levels at Klisoura cave in Greece [22] did not yield discernable ceramic “art,” but do suggest the emergence of a ceramic technology in the early Upper Palaeolithic. Similarly, ceramic fragments found in layers 1–2 at Kostenki I in Russia (radiocarbon dated to 21,930 years BP, ∼25,300 cal BP [23]) preserve cordage impressions [24] but were not appropriated to make art. Thus, the finds from Vela Spila seem to represent the first evidence of a developed Palaeolithic ceramic art technology and tradition that postdates the Last Glacial Maximum in Europe. The Vela Spila ceramics appear to be the result of an independent invention that is unrelated to the disparate ceramic technologies that precede it elsewhere in Europe.

No ceramics have been found in Mesolithic horizons at Vela Spila, so more than 8,000 years separate the Palaeolithic ceramics from the site’s earliest Neolithic ceramic pottery. The earliest Neolithic, or Impressed-ware ceramics at Vela Spila date to between *c.* 7,000–6,400 uncal BP. Impressed-ware vessels feature the use of shells, fingernails, or other implements to create patterned incisions and marks [25]. The impressions on C2 may, initially, seem similar to Impressed-ware, but several significant differences distinguish them. The C2 impressions are much smaller than the marks typically made on Impressed-ware vessels and more irregular than Impressed-ware incisions because different tools and implements were used. Whereas a stone tool, such as a burin, was probably used to incise C2, Impressed-ware pottery was marked with shells (most famously *Cardium* sp. shells), indicating the development of distinct cultural and technical traditions. Finally, the marks are not regularly arranged on C2, whereas Impressed-ware pottery features marks which are often evenly distributed or grouped in patterns. The similarities between the incisions covering C2 and those noted on Pavlovian ceramics reinforce the differences between the Palaeolithic and Neolithic ceramic traditions.

Early Neolithic ceramic figurines are rare in the Balkans. Only one such statuette is currently known from Croatia, which was found in Vela Spila in 2004. This Neolithic statuette, which might depict a pig, is somewhat larger than most of the Pleistocene ceramics (33.0×18.0×15.0 mm). One key techno-stylistic difference relates to the way legs were formed. Palaeolithic ceramicists combined zoomorphic legs in both C1 and C2 so that only two limbs were modeled, rather than four. However, the Neolithic figurine preserves four breaks, where four separate limbs were joined to the body. The Palaeolithic tradition of combining four legs into two is not expressed here, illustrating a different stylistic and technical convention in the Neolithic. Thus, the stratigraphic evidence and stylistic and technological differences between the Palaeolithic and Neolithic figurines reinforce the authencity of the new finds from Vela Spila as securely Palaeolithic. Furthermore, it suggests that the Palaeolithic ceramic tradition may have been considerably different from the later Neolithic ones that developed in this area, with no evidence of continuity between these two time periods. Indeed, Palaeolithic and Neolithic craftspeople and artists seem to have independently invented ceramics in two very different social contexts.

Despite some technological and stylistic similarities between the Pavlovian and Vela Spila ceramic assemblages, the technocomplexes share little other material culture suggesting cultural continuity spanning thousands of kilometers and 10,000 years. Pavlovian ceramic technology seems to have remained a geographically circumscribed tradition within Central Europe. Furthermore, Pavlovian ceramic artifacts disappear from the archaeological record only a few millennia after their first appearance, *c.* 27,000 cal BP, when the Pavlovian technocomplex either transformed into or was replaced by a later Gravettian culture (the so-called Willendorf-Kostenkian). The geographic, chronological, and techno-cultural differences between Gravettian Moravia and Epigravettian Croatia make it reasonable to purport an independent invention of this technology and tradition in the western Balkans almost 10,000 years later. Without destructive technological analysis of the materials used to make the ceramics at Vela Spila, it remains impossible to determine if the ceramics were made *in situ* at Vela Spila or if they were imported from elsewhere in the region. However, the lack of Pleistocene ceramics at contemporaneous sites in the region, such as Crvena Stijena (Montenegro), Kopačina (Croatia), Badanj (Bosnia and Herzegovina), and Grotta Paglicci (Italy), suggests the tradition may have developed at Vela Spila.

**Figure 11:**
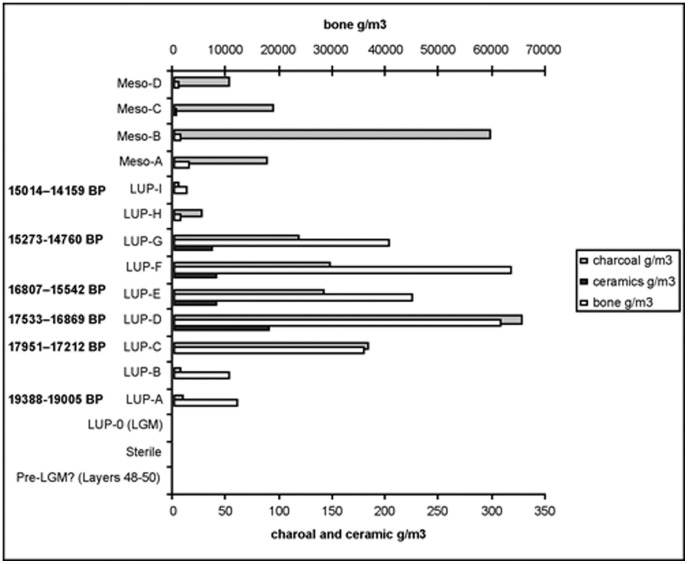
Concentrations of charcoal, ceramics and bone in Late Upper Palaeolithic and Mesolithic horizons at Vela Spila.

The stratigraphic sequence at Vela Spila demonstrates Upper Palaeolithic occupation as much as 2,000 years before the emergence of ceramics and also slightly after the ceramic figurines disappear from the record. Interestingly, charcoal and faunal remains are most abundant in horizons where ceramics were found (Figure 11). This period of increased activity may have encouraged innovation, including the invention and use of ceramic technologies. Thus, as in Gravettian Moravia, ceramic technology appears to have been invented in the southwest Balkans, used for an extended period of time, and then it seems to have been either forgotten, rejected or replaced by another technology. Ceramic technologies do not re-emerge in the sequence at Vela Spila until the Neolithic, when the material was used primarily to make functional pottery rather than representational figurines.

The ornaments and decorated bones from Vela Spila suggest that a symbolic tradition existed here throughout much of the late Upper Palaeolithic. Perforated marine shells and red deer canine ornaments at the site are similar in form throughout the late Upper Palaeolithic sequence, indicating a stable decorative or symbolic tradition. The raw materials were minimally modified to make the ornaments; in most instances, a single perforation was made in the material. The stratigraphic sequence demonstrates that the ornaments predate the ceramic technologies; when ceramics begin to appear in the record, they are often found associated with these ornaments in the same layers. After the ceramics disappear from the record, shell and tooth ornaments continue to be found. Figurative engravings or sculptures made in bone, antler, or stone, similar to those found in post-LGM contexts elsewhere in Europe (e.g. the Magdalenian anthropomorphic sculptures and engravings from Rhineland Germany) have not been found at Vela Spila. Consequently, the two ceramic figurines discussed above and the purported leg fragments are the only evidence of Epigravettian figurative art at Vela Spila. Indeed, at Vela Spila, figurative art directly and exclusively correlates with the use of ceramics. Moreover, the exclusive use of ceramics for the production of figurative art at Vela Spila suggests artistic motivation for this innovation. The development of a new material and innovative technologies may have been a catalyst for transformation in artistic expression and the earliest figurative art at the site. Although Palaeolithic archaeologists often focus on “functional” or “utilitarian” innovations as important moments of social transformation (e.g. fire, Levallois technology, bone tool manufacture), the ceramics from Vela Spila offer a glimpse into the ways a symbolic innovation can significantly alter the scope of artistic expression within a culture.

### Conclusions

The ceramic figurines and fragments from Vela Spila are the first evidence of a developed artistic ceramic technology and tradition in Europe after the LGM. These new finds encourage reconsideration of the conventional conception of ceramics as a primarily Neolithic technology. Furthermore, they offer support for the notion that ceramics were first used to make art rather than functional or utilitarian material culture such as vessels. This association of the earliest iteration of the innovative material with ornamental or decorative artifacts echoes the development of metallurgy, which also often emerged first in non-utilitarian contexts [26]. Indeed, the Vela Spila ceramics demonstrate that several distinct Palaeolithic societies made art from ceramic materials more than 10,000 years before the earliest evidence of ceramics and pottery in the Neolithic. Additionally, as the only evidence of figurative art from this site, these artifacts offer important insight into an emerging representational art tradition in the Balkans *c.* 17,500 years ago.

Both stylistically and technologically, the figurines from Vela Spila recall the Pavlovian ceramic figurines from Moravia. Some similarities between Pavlovian figurative art and the Vela Spila ceramics may suggest that inherent qualities of this material contributed to the stylistic character of figurines made in ceramic. Compressed legs and the fragmentation of limbs and extremities in accordance with the additive method of production are found in figurines from both assemblages. However, differences in their spatial distribution pattern, as well as the geographic and chronological distance between southern Dalmatia and Moravia, suggest the emergence of a distinct socio-technical tradition during the Epigravettian in Croatia, *c.* 17,500-15,000 cal BP. This new evidence indicates that the Pavlovian ceramics are not a unique Palaeolithic technology. Rather, a variety of social, geographic, environmental, and chronological contexts over extended periods of time in Europe supported experimentation with ceramic to make art well before societies became more sedentary at the beginning of the Neolithic. Broadening our understanding and awareness of these materials in Palaeolithic contexts may increase the recovery of these idiosyncratic, but significant, artifacts during future excavations.
